# Shape-matching soft mechanical metamaterials

**DOI:** 10.1038/s41598-018-19381-3

**Published:** 2018-01-17

**Authors:** M. J. Mirzaali, S. Janbaz, M. Strano, L. Vergani, A. A. Zadpoor

**Affiliations:** 1 Department of Mechanical Engineering, Politecnico di Milano, Via La Masa 1, 20156 Milano, Italy; 2 Department of Biomechanical Engineering, Faculty of Mechanical, Maritime, and Materials Engineering, Delft University of Technology (TU Delft), Mekelweg 2, 2628 CD Delft, The Netherlands

## Abstract

Architectured materials with rationally designed geometries could be used to create mechanical metamaterials with unprecedented or rare properties and functionalities. Here, we introduce “shape-matching” metamaterials where the geometry of cellular structures comprising auxetic and conventional unit cells is designed so as to achieve a pre-defined shape upon deformation. We used computational models to forward-map the space of planar shapes to the space of geometrical designs. The validity of the underlying computational models was first demonstrated by comparing their predictions with experimental observations on specimens fabricated with indirect additive manufacturing. The forward-maps were then used to devise the geometry of cellular structures that approximate the arbitrary shapes described by random Fourier’s series. Finally, we show that the presented metamaterials could match the contours of three real objects including a scapula model, a pumpkin, and a Delft Blue pottery piece. Shape-matching materials have potential applications in soft robotics and wearable (medical) devices.

## Introduction

Mechanical metamaterials are materials whose macro-scale properties such as unusual deformation characteristics directly originate from their (small-scale) geometrical design^1–4^. Rational geometrical design of metamaterials could lead to properties and functionalities not usually offered by natural materials such as negative Poisson’s ratio (auxetics)^5–7^, negative compressibility^4^, elastic hysteresis^8^, independent tailoring of elastic properties^9^, snapping deformations^10^, and shape-changing with vibration-mitigation capability^11^ and out-of-plane deformation through 3D design of architectured metamaterials^12^.

Metamaterials have also potential applications in shape-changing materials that are kinematically inspired by kirigami/origami-based designs^13^, fractal cuts^14^, deployable morphing^15^, pattern switching^16^, or strain amplification elements working through auxetic unit cells^17^. A novel objective in the design of shape-changing metamaterials is achieving a pre-defined shape upon loading through what we here call “shape-matching” materials.

Shape-matching metamaterials have a myriad of potential applications most notably in soft robotics^18,19^, and wearable (medical) devices. For example, shape-matching metamaterials could be used to design soft grippers that grip delicate objects with the maximum surface contact and, thus, minimum contact force. Wearable (medical) devices such as exosuits^20^, prosthetics and orthotics^21^, and tunable mechanical memory^22^ are the other potential areas of application. Finally, the fashion design industry^23^ may also be able to benefit from shape-matching or form-fitting materials.

**Figure 1:**
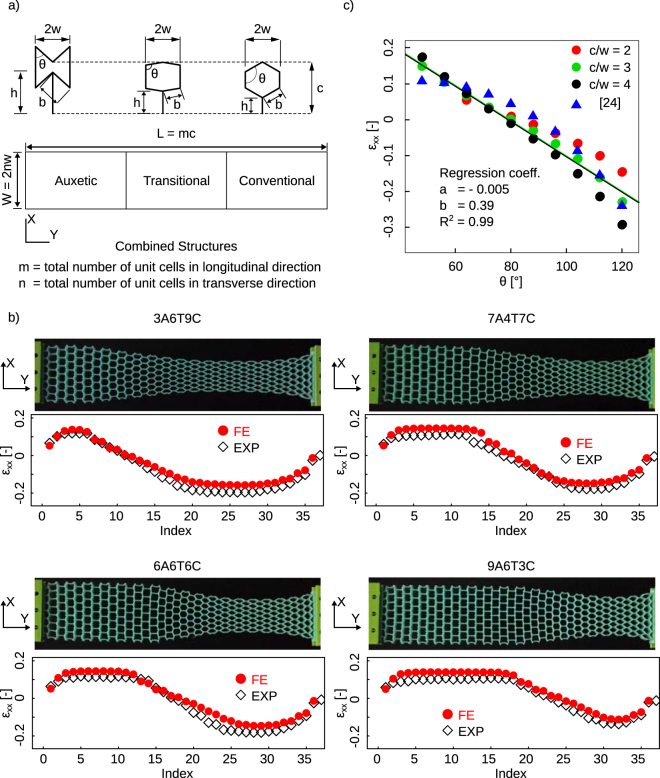
(**a**) A schematic drawing of the auxetic and conventional unit cells. The angle, *θ*, varies between 48° and 120°. (**b**) Four specimens with three zones (auxetic, transition and conventional) were fabricated. Computational models were developed for each specimen and the simulation results were compared against experimental observations. The specimen naming convention follows the NoANoTNoC format where “No” shows the number of unit cells in each region, while A, T, and, C stand for the number of unit cells in the auxetic, transitional, and conventional regions. In the transitional region, unit cells were linearly changed from auxetic to conventional unit cells. In all experimental specimens, *c*/*w* = 3. Index refers to the points at the corner of each unit cell where the lateral strains were calculated. (**c**) The strains of cellular structures calculated for different c/w ratios and reference angles (longitudinal strain = 20%). Numerical results were also compared with the analytical relation (Equation 2) in the literature^24^ for the calculation of lateral strains.

Here, we demonstrate soft shape-matching metamaterials that are designed by rationally combining auxetic, conventional, and transitional unit cells into a cellular solid, and are indirectly additively manufactured from elastomers (Fig. 1). We used an equal aspect ratio for the longitudinal and transversal dimensions of all unit cells for easier integration of the unit cells with different reference angles in a planar cellular structure. This enables us to combine auxetic and conventional unit cells in the longitudinal direction, which could be also replicated in the transverse direction. The arrangement of unit cells with different values of the Poisson’s ratio could then be used to program the lateral deformation of the cellular material upon deformation. The inverse problem of rationally designing a shape-matching metamaterial then reduces to the problem of finding the combinations of the Poisson’s ratios that give rise to the desired lateral deformation and mapping those values of the Poisson’s ratios back to unit cell designs.

## Methods

To design the first prototypes, we divided the length of the specimen into three regions, i.e., auxetic, transitional, and conventional (Fig. 1a). When designing the cellular structures, we assumed that the parameters *c* and *w* (Fig. 1a) are constant for both auxetic and conventional unit cells $(c/w=3)$. The interior angle of each unit cell could therefore change the geometry of the unit cell from auxetic $(48^\circ  < \theta  < 90^\circ )$ to conventional $(90^\circ  < \theta  < 120^\circ )$. In the transitional region, the angle of each unit cell linearly changed from the auxetic angle to conventional one. We fabricated four prototypes with different unit cells in each of those three regions. The total numbers of unit cells in the longitudinal direction, *m* = 18, was similar for all specimens. The total length of each specimen was therefore *L* = *c* × *m*. The number of unit cells in the transverse direction, *n*, was also fixed (*n* = 7). The width of each specimen was therefore *W* = 2 × *n* × *w*. The design parameters for all specimens are presented in Table 1.

**Table 1:** The parameters of the prototypes fabricated with indirect additive manufacturing.

parameters	*c*[*mm*]	*w*[*mm*]	*n*[−]	*m*[−]	*L*[*mm*]	*W*[*mm*]	*t*_*lattice*_[*mm*]	*t*_*ligament*_[*mm*]
	15	5	7	18	270	70	7	0.7

We used indirect additive manufacturing to fabricate the specimens. A mold was designed and additively manufactured using a fused deposition modeling (FDM) 3D printer (Ultimaker 2+, Geldermalsen, The Netherlands) from polylactic acid (PLA) filaments (MakerPoint PLA 750 gr Natural). Subsequently, an elastomeric polymer (Elite Double 8, Zhermack, Badia Polesine, Italy) with a one-to-one ratio of the base to catalyst was poured into the mold. Once the specimens had cured, i.e., after ≈1 hour, the mold was removed. The final shapes of all specimens are presented in Fig. 1b.

The specimens were deformed under displacement-controlled tensile loading applied by a mechanical test bench (LLOYD instruments, LR5K, load cell = 100 N, displacement rate = 50 mm/min). Time, force, and displacement were recorded at a sampling rate of 20 Hz. Simultaneously, the deformation was captured by a digital camera. After the test, the first and last frames of each video was selected and converted into binary images. The middle junctions in each unit cell at the left and right sides of the structure were highlighted by points in the images. Then, the highlighted regions were dilated to one pixel. The initial width of each unit cell, *W*_*j*_, was calculated from the first image as: ${W}_{j}={X}_{j,right}-{X}_{j,left}$, where *j* = 1:36 shows the number of points in the total length of the structure, also called as index, and *X*_*j*_ stands for the position of each point measured in terms of pixels. Every two points belong to one unit cell. Similarly, after deformation, the final width of each unit cell, ${W}_{j}^{\text{'}}$, was calculated as ${W}_{j}^{\text{'}}={X}_{j,right}^{\text{'}}-{X}_{j,left}^{\text{'}}$. Finally, the lateral strain was defined as ${\varepsilon }_{j,xx}=\frac{{W}_{j}^{\text{'}}}{{W}_{j}}$.

Computational models of the metamaterials were created with ABAQUS, 6.14. A hyperplastic Neo-Hookean material model (${C}_{10}=0.106\,MPa\,\,\,$and ${D}_{1}=0.03MP{a}^{-1}$) and plane-stress elements (CPS8) were used in the models. The material coefficients were determined using the standard experimental protocols for testing elastomeric materials in tension (ASTM D412 Type C) and compression (ASTM D 575–91). The out-of-plane thickness, *t*_*lattice*_, of the structure and the thickness of each ligament, *t*_*ligament*_ were respectively set to 10 mm and 1 mm. Two reference points were defined at the top and bottom of the model and were tied to two nodes from the corresponding locations. The bottom reference point was fixed while the top reference point was displaced far enough to create 20% longitudinal strain. An implicit nonlinear solver (Abaqus Standard) was used for the simulations. The lateral strain calculated for the computational models were evaluated in a way similar to those of the experiments. Several node sets were defined at the internal hinges of the unit cells located at the left and right sides of the structure. The mean lateral displacements ${U}_{j,left},{U}_{j,right}$ were calculated for each node set. The lateral strain was then obtained as: ${\varepsilon }_{j,xx}=\frac{{U}_{j,right}-{U}_{j,left}}{W}$.

The lateral strains obtained computationally were found to be in good agreement with experimental observations (Fig. 1b). Having evaluated the accuracy of the computational models, we expanded them to include other *c*/*w* ratios, i.e. 2, and 4, for fully auxetic and conventional lattice structures (Fig. 1c). In those simulations, the total number of unit cells in the structure was kept the same to enable comparison with previous results and the lateral strain was calculated at the middle of the cellular structure. Our calculations showed changing this ratio does not drastically change the lateral strains especially for the auxetic unit cells (Fig. 1c). Therefore, we continued to use $c/w=3$ as a reference ratio in the following designs. Furthermore, the computational results showed an almost linear relationship between the lateral strains and the angle of the unit cells (Fig. 1c). This linear relation was used as the basis of our designs in the next steps, where the applied longitudinal strain was fixed at 20%:

1 $${\varepsilon }_{xx}=-0.005\theta +0.39$$

We compared our numerical simulations with the analytical relation found in the literature^24^. Lateral strain in the conventional and the re-entrant honeycombs can be calculated as:

2 $${{\rm{\varepsilon }}}_{{\rm{x}}}=-\frac{{\rm{\delta }}\,\sin \,{\rm{\theta }}}{{\rm{b}}\,\cos \,{\rm{\theta }}}$$

where, ${\rm{b}}$ and ${\rm{\theta }}$ are the geometrical parameters of each unit cell (Fig. 1a) and ${\rm{\delta }}=\frac{0.2}{\cos \,{\rm{\theta }}}({\rm{c}}-{\rm{h}})$ as we fixed the applied longitudinal strain at 20%. The geometrical parameters, i.e. ${\rm{b}},\,{\rm{\theta }},\,{\rm{c}}$ and h, were considered separately for individual unit cells and the final lateral strains were compared with the numerical simulations. This comparison shows a good agreement between the numerical and analytical results (Fig. 1c).

We also performed experiments to evaluate the effects of the number of unit cells in the transverse direction of the structure on the lateral deformation (see the Supplementary Materials). We found that the number of the unit cells in the transverse direction only influences the amplitude of the deformations but not the lateral strains of the structure.

## Results and Discussion

**Figure 2:**
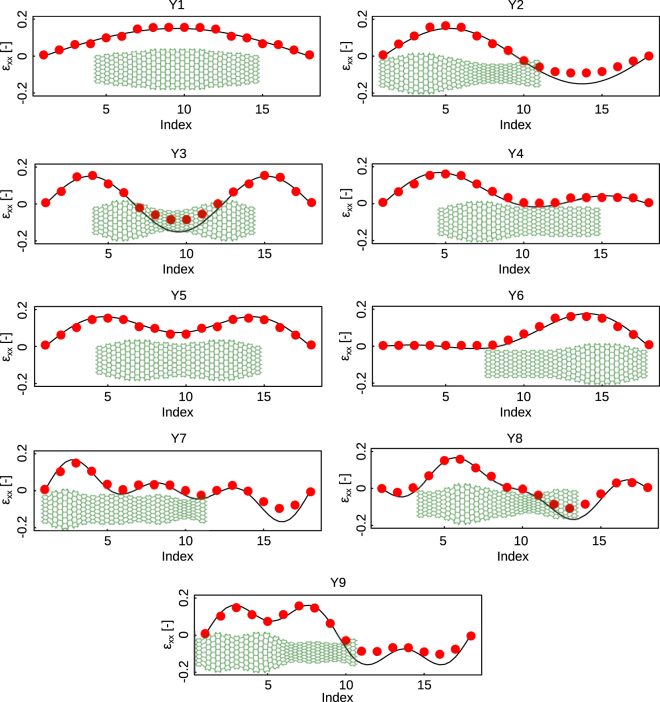
The structures were designed in a way to fit the arbitrary functions (Y1–Y9). The parameters of the functions are listed in Table 2. The longitudinal strain for each case is equal to 20% of the total length. The actual deformations determined using direct numerical simulations are also shown (scaling factor = 3). Index refers to the middle point of each unit cell where the lateral strain is calculated.

Having verified our computational models against experiments for a number of designs, we developed a rational design platform based on the results of the computational models to achieve lateral deformations that match the contour of an arbitrarily-shaped object. If the contour of the lateral deformation is discretized into a finite number of sub-regions, auxetic and conventional unit cells could be used to create the desired lateral deformation using Equation (1). The superposition of the deformations of all unit cells was hypothesized to create the target shape. To assess the validity of that hypothesis, we created a number of arbitrarily-defined strain functions (Y1–Y9) using a three-terms Fourier’s-like series:

3 $$Y={a}_{1}\,\sin (\alpha \omega (x-1))+{a}_{2}\,\sin (2\alpha \omega (x-1))+{a}_{3}\,\sin (3\alpha \omega (x-1))$$

where the parameters *ω* = 0.37 and ${a}_{1},\,{a}_{2},\,{a}_{3},\,\alpha$ (Table 2) were parametrically selected in order to achieve substantially different mode of deformations and covering a wide range of deformed shapes (Fig. 2). *x* is the index of each unit cells in the longitudinal direction and changes between 1 and 18 (the total number of unit cells in the longitudinal direction of the cellular structure). Using Equation (1), we selected the angles of the auxetic and conventional unit cells such that their predicted deformation would follow the strain functions generated by Equation (3) using the above-mentioned coefficients (Fig. 2). We then created computational models to determine the actual deformation of the cellular structures that were designed using the predictions of Equation (1). Comparison between the actual deformations and the target strain functions showed that the cellular structures designed using Equation (1) could closely follow the target shape in all considered cases (Fig. 2).

**Table 2:** The random parameters used in the definition of the functions (Y1–Y9).

Functions	*a*_1_	*a*_2_	*a*_3_	*α*
Y1	0.15	0	0	0.5
Y2	0	0.15	0	0.5
Y3	0	0	0.15	0.5
Y4	0.068	0.068	0.068	0.5
Y5	0.15	0	0.075	0.5
Y6	0.075	−0.09	0.045	0.5
Y7	0.068	0.068	0.068	1
Y8	0.105	−0.053	−0.053	1
Y9	0.15	0	0.075	1

**Figure 3:**
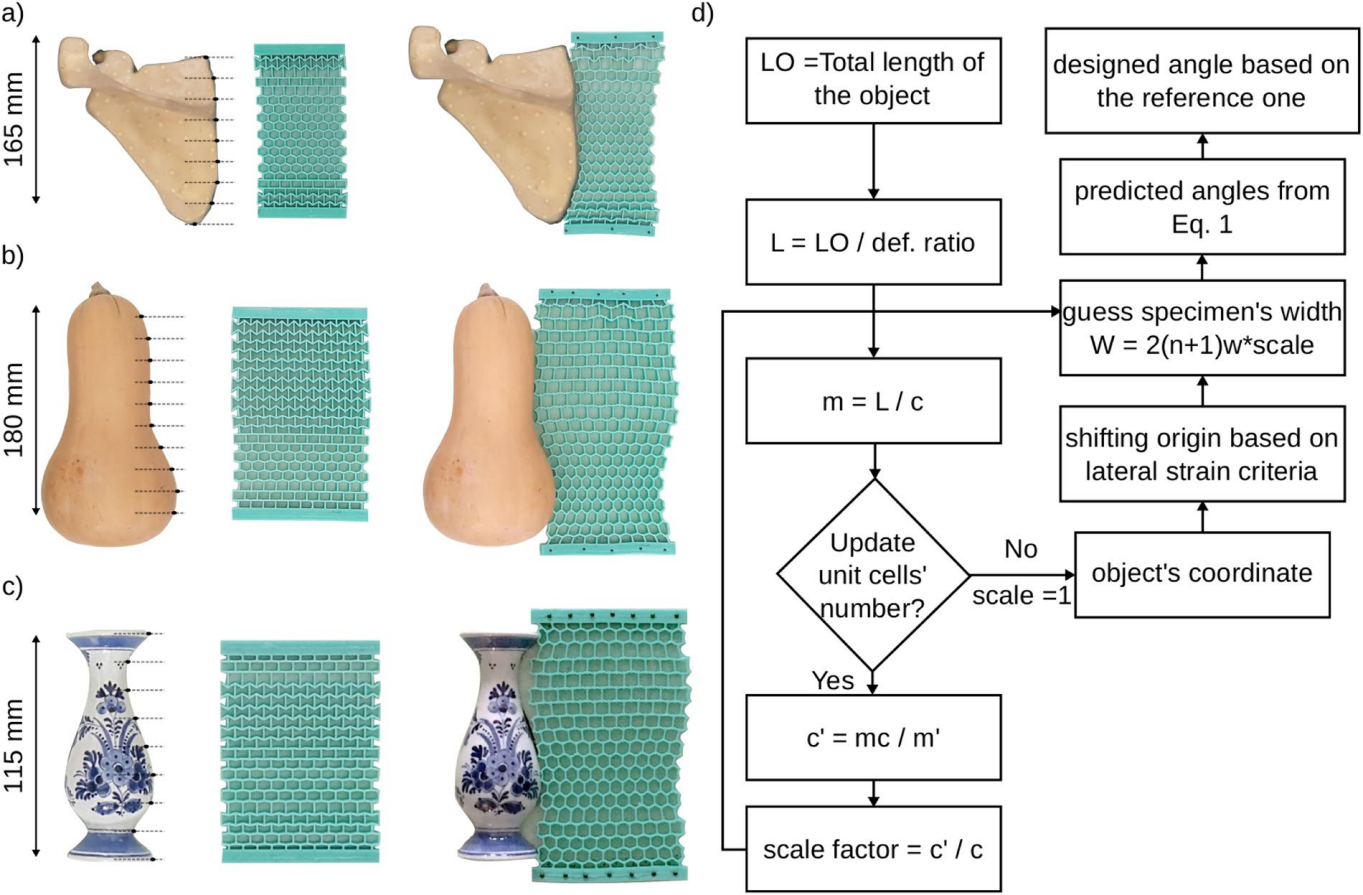
Matching the shapes of three real objects including an anatomical model of the scapula (**a**), a pumpkin (**b**), and a Delft Blue pottery piece (a vase) (**c**). (**d**) The flowchart of the design procedure.

In the last step, we aimed to design cellular structures that would match the shapes of three real objects including an anatomical model of the scapula (Sawbones, Vashon Island, USA), a pumpkin, and a piece of Delft Blue pottery (a vase) (Fig. 3). All objects were photographed from the top view, which was then used to describe the contours of the objects. The resulting contours then served as the target shapes for the design of the cellular structures. We selected the reference angles of the auxetic and conventional unit cells such that, according to Equation (1), their lateral deformations match the captured contours as closely as possible. The total number of unit cells in the transverse and longitudinal directions are therefore the only parameters that could be freely chosen with more unit cells along the length of the structure resulting in smoother approximations of the target curve. To use Equation (1), the design of the cellular structure needs to satisfy $c/w=3$, which could be achieved by isotropic scaling of the entire structure. Assuming that we require a 20% deformation of the metamaterial to match the shape of the objects, the length of the cellular structures could be determined using a deformation ratio of 1.2. Given the total length of the specimen and the parameter *c*, the maximum number of unit cells along the length of the structure, *m*, was calculated. Having assumed the number of unit cells in the transverse direction, *n* + 1, the lateral strain could be calculated, which must be in the range of the minimum and maximum strains that fully auxetic and conventional structures could achieve. Finally, the reference angles of the unit cells were selected using Equation (1). A flowchart in Fig. 3d shows the different design steps.

The cellular structures designed to match the shapes of the selected objects achieved reasonable approximations of their target contours (Fig. 3a–c). The shapes of the objects used here were selected based on the potential applications of shape-matching metamaterials. Moreover, each object represented different types of shape variations. For instance, the contour of the pumpkin requires the highest deformation amplitude. To achieve such high amplitudes, it is necessary to define more unit cells in the lateral direction of the structure (Fig. 3b). The vase, on the other hand, has the smallest length among three objects. Therefore, application of a scaling factor is required when designing the shape-matching metamaterial (Fig. 3c).

The fact that a simple equation such as Equation (1) and the superposition principle are very effective in designing soft metamaterials (whose deformation is nonlinear in nature) is quite remarkable and enables the fast design of shape-matching metamaterials without the need for (nonlinear) optimization algorithms. The presented technique could be expanded for the prediction of the deformations at lower (i.e., micro-) scales. While we used here mechanical loading to deform the specimens, shape-matching could also be activated using magnetic, thermal, or electrical stimuli. Shape memory polymers are the other candidates for such designs^25–28^.

There are some limitations in the presented approach that are dictated by the maximum strains that could be achieved by conventional and auxetic unit cell. In general, auxetic unit cells tend to deform faster than the conventional ones and reach a level of saturation and robustness at higher longitudinal strains. This affects the connection between conventional and auxetic unit cells and prevents the conventional unit cells from complete deformation (see the Supplementary Information). For this reason, we fixed the level of axial strain to 20% so that we will ensure different unit cells can reach their maximum expansion without being affected by the adjacent unit cells.

The next steps would entail extending the presented technique to the case of three-dimensional shapes such as the shapes described by the surface of the human body. One way of creating 3D structure is rolling the proposed 2D lattice structures to create tube-like structures. The shape-matching behavior of such axisymmetric structures is expected to be similar to those of 2D structures. For arbitrary 3D shapes, similar discretization method on surfaces rather than lines can be applied, although the complexity of the problem will increase due to the unknown interaction of individual unit cells. Direct 3D printing with similar elastomeric polymers could be used for the production of such 3D structures.

In summary, we presented a design platform for the rational design of shape-matching soft mechanical metamaterials that combine functionally graded auxetic and conventional unit cells. The platform is shown to be able to match the arbitrary shapes created by three-term Fourier’s series as well as the shape of real objects. Shape-matching materials have potential applications in soft robotics, wearable (medical) devices, and fashion industry.

## Electronic supplementary material

Supplementary document
