# Supplementary material for: Shape-matching soft mechanical metamaterials
Source: Sci Rep. 2018 Jan 17;8:965. doi: 10.1038/s41598-018-19381-3 (PMC5772660; doi:10.1038/s41598-018-19381-3)
Supplement: Supplementary file 1 — Supplementary document [file 41598_2018_19381_MOESM1_ESM.doc]

Supplementary document to

Shape-matching soft mechanical metamaterials

M. J. Mirzaali^a,b,c^,[[1]](#footnote-2), S. Janbaz^b,c^,[[2]](#footnote-3), M. Strano^a^, L. Vergani^a^, A.A. Zadpoor^b^

*^a^Department of Mechanical Engineering, Politecnico di Milano, Via La Masa 1, 20156 Milano, Italy*

*^b^Department of Biomechanical Engineering, Faculty of Mechanical, Maritime, and Materials Engineering, Delft University of Technology (TU Delft), Mekelweg 2, 2628 CD, Delft, The Netherlands*

Pure auxetic and conventional lattice structure were experimentally tested (Figure S1). Auxetic unit cells reached the saturation levels of strains earlier than conventional unit cells. The maximum level of lateral strain that could be reached with fully conventional structures is lower than the combined structures.

**
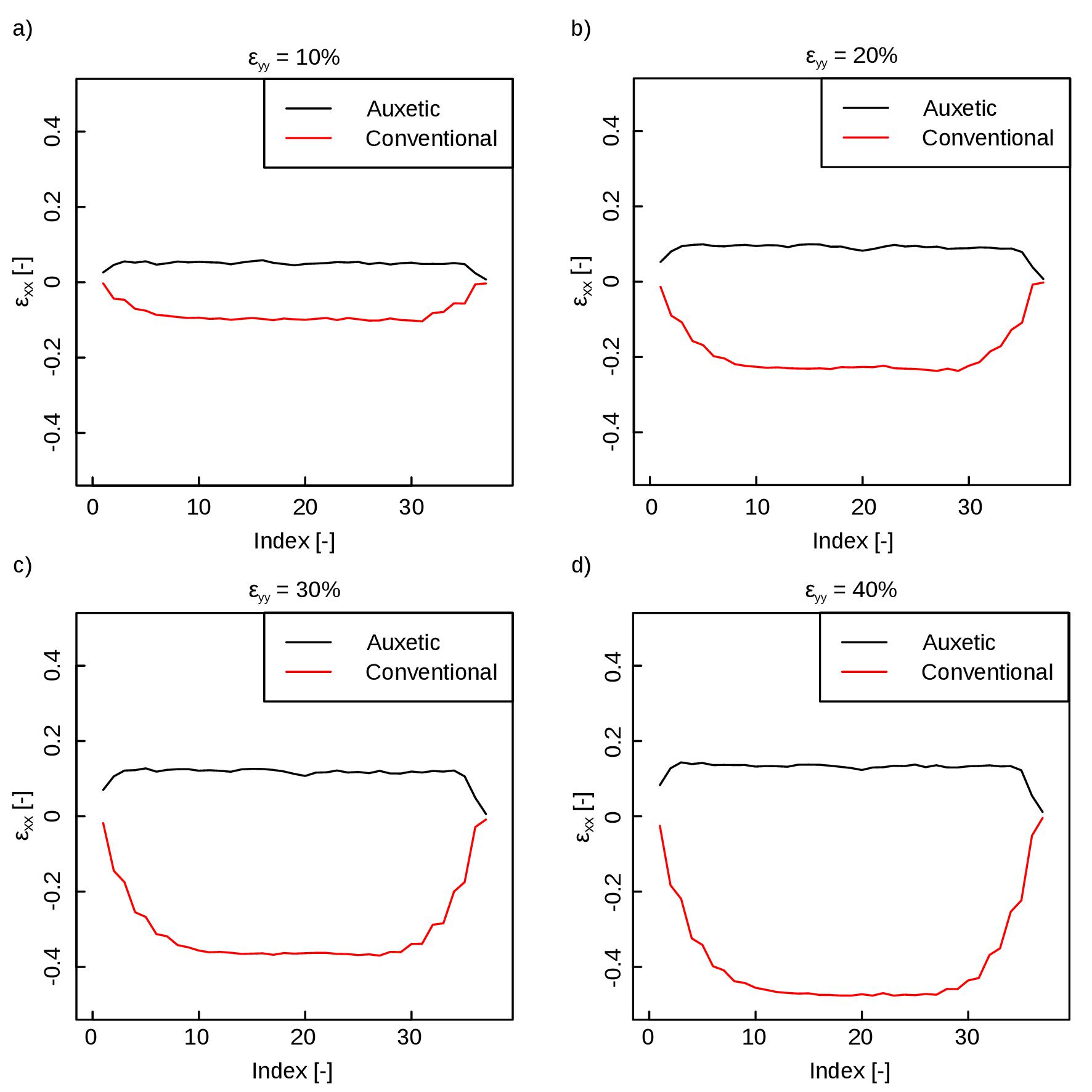
**

**Figure S1.** Lateral strain at four different longitudinal strains for pure auxetic and pure conventional lattice structures. The angle of auxetic and conventional structures were 48 and 120, respectively.

The total number of unit cells in the transverse direction does not influence the lateral strains. This is shown from the experiments Figure S2.

**
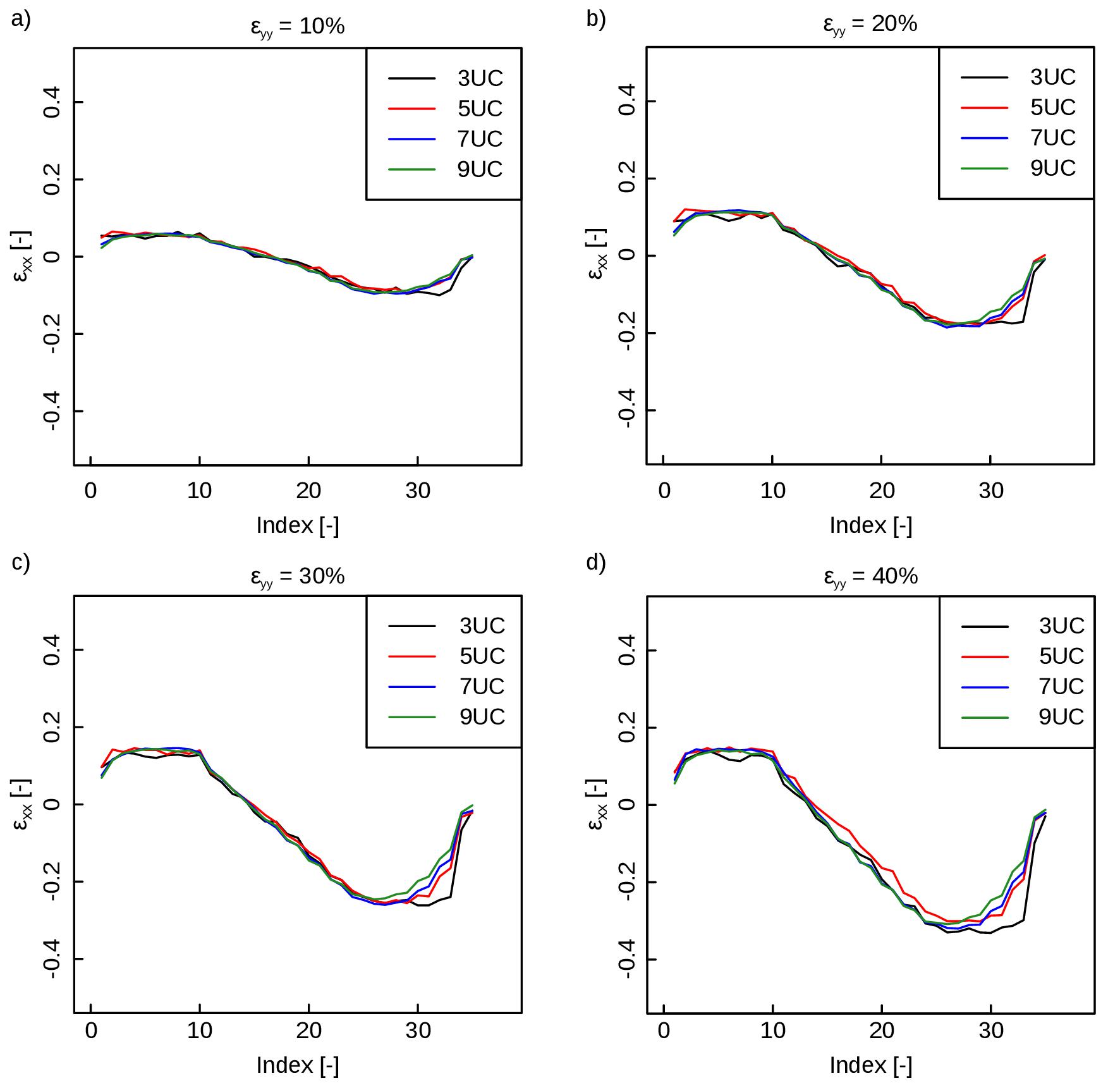
**

**Figure S2.** The effects of the number of unit cells in the transverse direction of combined structure on the lateral strains. Results are plotted at four extension levels (a-d). Equal number of conventional, auxetic and transitional unit cells were considered for the construction of these specimens.

Considering the experimental results for the combined structures, it was observed that changing the regions (auxetic, transition, conventional) in the structure does not influence the maximum lateral strains in the auxetic unit cells. However, due to the geometrical features of the conventional unit cells, they were more sensitive to the boundary conditions and deformation of the adjacent unit cells. In the transitional region, we found that lateral strain linearly changes along the length (Figures S3 and S4).

**
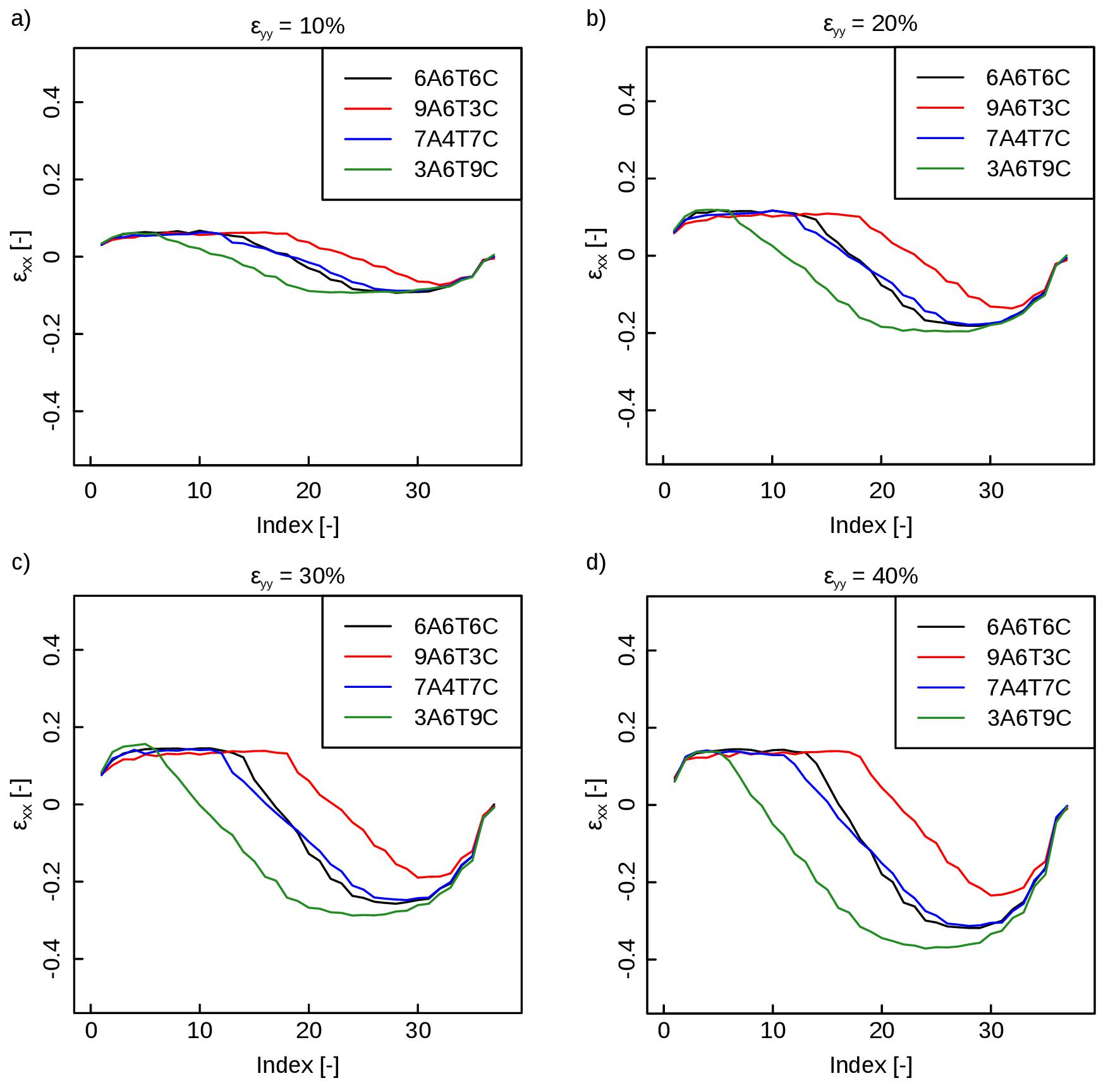
**

**Figure S3**. Comparison of lateral strains at different levels of longitudinal expansion for four combined specimens. The specimen naming convention follows the NoANoTNoC format where “No” shows the number of unit cells in each region, while A, T, and, C stand for the number of unit cells in the auxetic, transitional, and conventional regions. In the transitional region, unit cells were linearly changed from auxetic to conventional unit cells. In all experimental specimens, *c*/*w*=3.


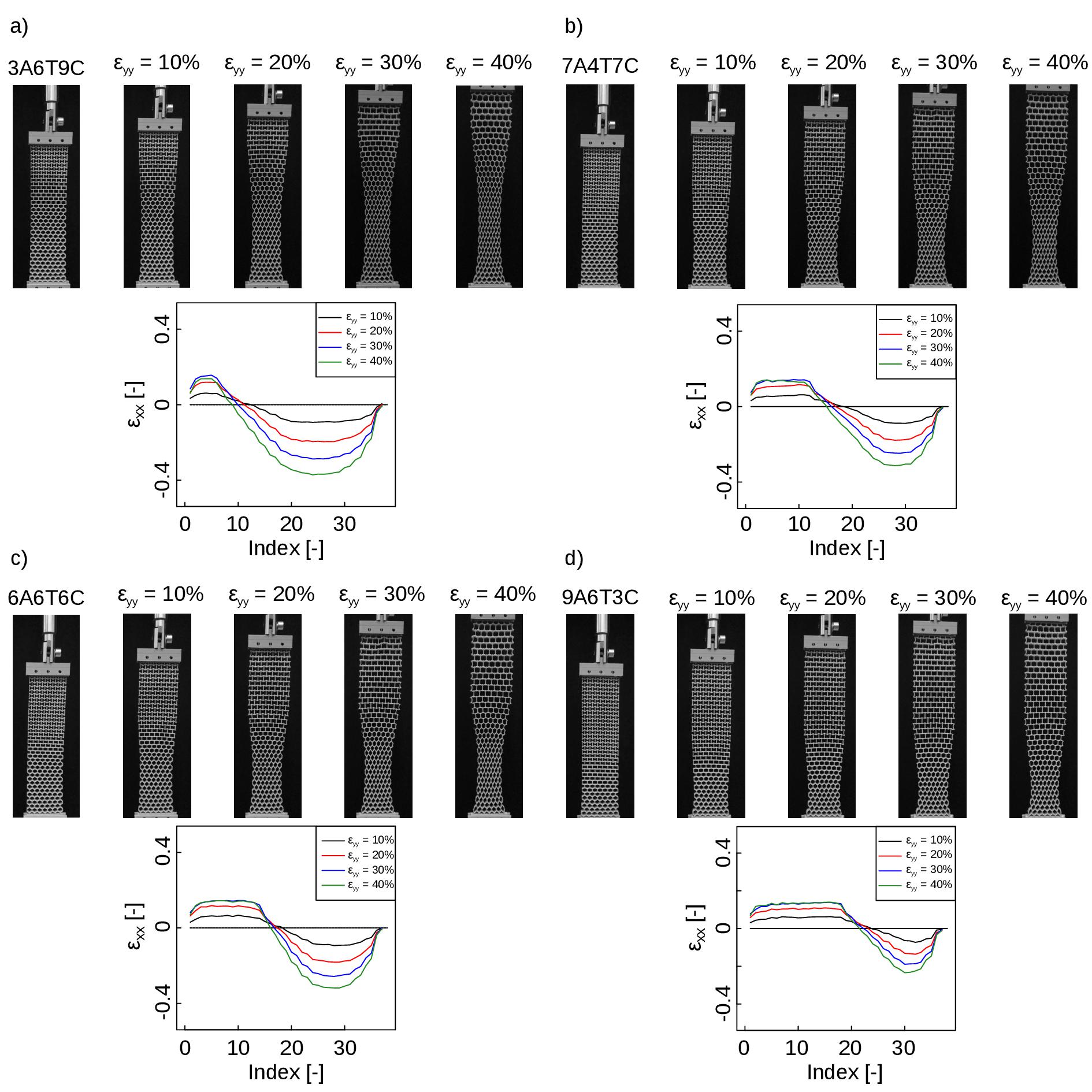


**Figure S4.** Individual lateral strains at different levels of longitudinal expansions for four combined specimens. a) 3A6T9C, b) 7A4T7C, c) 6A6T6C, d) 9A6T3C. The specimen naming convention follows the NoANoTNoC format where “No” shows the number of unit cells in each region, while A, T, and, C stand for the number of unit cells in the auxetic, transitional, and conventional regions. In the transitional region, unit cells were linearly changed from auxetic to conventional unit cells. In all experimental specimens, *c*/*w*=3.

1. *E-mail address:* [mirzaalimazandarani.mohammad@polimi.it](mailto:mirzaalimazandarani.mohammad@polimi.it); [mirzaali.mohammad@gmail.com](mailto:mirzaali.mohammad@gmail.com)

   ^C^ Both authors share first authorship. [↑](#footnote-ref-2)
2. Corresponding author. Tel.: +31-15-2783133.

   *E-mail address:* [s.janbaz@tudelft.nl](mailto:s.janbaz@tudelft.nl); [shahram.janbaz@gmail.com](mailto:shahram.janbaz@gmail.com)

   ^C^ Both authors share first authorship. [↑](#footnote-ref-3)
